# Insights into Conformational Dynamics and Allostery in DNMT1-H3Ub/USP7 Interactions

**DOI:** 10.3390/molecules26175153

**Published:** 2021-08-25

**Authors:** Yu Zhu, Fei Ye, Ziyun Zhou, Wanlin Liu, Zhongjie Liang, Guang Hu

**Affiliations:** 1 Center for Systems Biology, Department of Bioinformatics, School of Biology and Basic Medical Sciences, Soochow University, Suzhou 215123, China; yzhu99@stu.suda.edu.cn (Y.Z.); zhouziyun1900@hotmail.com (Z.Z.); LiuWanlin1998@163.com (W.L.); 2 College of Life Sciences and Medicine, Zhejiang Sci-Tech University, Hangzhou 310018, China; yefei@zstu.edu.cn

**Keywords:** DNA methylation, DNMT1, protein-protein interactions, allosteric communication, drug design

## Abstract

DNA methyltransferases (DNMTs) including DNMT1 are a conserved family of cytosine methylases that play crucial roles in epigenetic regulation. The versatile functions of DNMT1 rely on allosteric networks between its different interacting partners, emerging as novel therapeutic targets. In this work, based on the modeling structures of DNMT1-ubiquitylated H3 (H3Ub)/ubiquitin specific peptidase 7 (USP7) complexes, we have used a combination of elastic network models, molecular dynamics simulations, structural residue perturbation, network modeling, and pocket pathway analysis to examine their molecular mechanisms of allosteric regulation. The comparative intrinsic and conformational dynamics analysis of three DNMT1 systems has highlighted the pivotal role of the RFTS domain as the dynamics hub in both intra- and inter-molecular interactions. The site perturbation and network modeling approaches have revealed the different and more complex allosteric interaction landscape in both DNMT1 complexes, involving the events caused by mutational hotspots and post-translation modification sites through protein-protein interactions (PPIs). Furthermore, communication pathway analysis and pocket detection have provided new mechanistic insights into molecular mechanisms underlying quaternary structures of DNMT1 complexes, suggesting potential targeting pockets for PPI-based allosteric drug design.

## 1. Introduction

**Figure 1:**
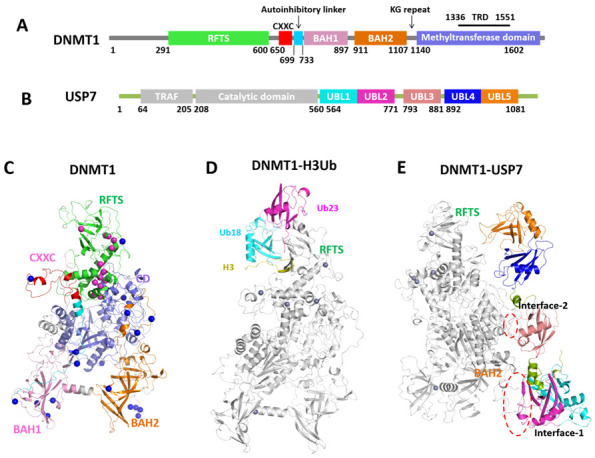
Structures of DNMT1 and its complexes with different partner proteins. (**A**) Color-coded domain architecture and numbering of human DNMT1 sequence. (**B**) Color-coded domain architecture and numbering of human USP7 sequence. (**C**) DNMT1 structure colored by subdomains, while disease mutations and PTM sites are denoted as magenta and blue spheres, respectively. (**D**) The constructed structures of DNMT1-H3Ub, DNMT1, Ub18, Ub23, and the H3 tail are colored grey, cyan, magenta, and yellow, respectively. (**E**) The constructed structure of DNMT1-USP7 and DNMT1 is colored grey and USP is colored by different modules.

As an important member of DNA methyltransferases (DNMTs), DNMT1 is involved in many human diseases through aberrant DNA methylation, such as cancers and neurological disorders, thus representing a promising therapeutic target [1,2]. From the structural point of view (Figure 1A,C), DNMT1 is a multidomain protein machine consisting of an N-terminal platform and a catalytic C-terminal domain (CD) [3,4]. The regulatory N-terminal platform includes the replication foci targeting sequence (RFTS) domain, a zinc-finger-like (CXXC) motif, two bromo-adjacent homology (BAH1 and BAH2) domains, and a flexible linker composed of lysine-glycine (KG) repeats [5]. For such a molecular machine, its biological functions depend on allosteric communication between the regulatory N-terminal regions and CD. To this aim, our previous studies have revealed the detailed regulatory mechanisms of DNMT1: (1) the conformational changes in the catalytic helix (residues 1227–1243) between different DNMT1 states allosterically regulate the enzymatic activity [6]; and (2) the domain-based allostery model suggested that the RFTS domain acted as an allostery “hub” to mediate the inter-domain signal transmission through the TRD interface [7]. However, we have explored only a minuscule fraction of their allosteric regulations.

DNMT1 can interact with wide and diverse regulatory partners as well as downstream effectors to perform its critical functions in cellular signaling. It was reported that DNMT1 alone cannot perform its methylation function during somatic cell divisions [8]. The UHRF1 (ubiquitin-like with PHD and ring finger domains 1) protein, which is in contact with the RFTS domain, recruits DNMT1 to DNA replication foci and is critical for DNA methylation [9,10]. The C-terminal RING domain of UHRF1 functions as an E3 ubiquitin ligase to ubiquitinate histone H3 [11,12], which is subsequently recognized by the RFTS domain of DNMT1 [9,10]. This process is important for the proper subnuclear localization of DNMT1 and maintenance of DNA methylation [13,14]. The crystal structure of the RFTS domain in complex with ubiquitylated H3 (H3Ub) was resolved, in which the ubiquitin-interacting motif within the RFTS was identified as an H3Ub binding region [14]. Besides ubiquitination governed by UHRF1, the stability, function, and abundance of DNMT1 during the cell cycle are also dependent on deubiquitination via the ubiquitin specific peptidase 7 (USP7), which protects DNMT1 from proteasomal degradation. The crystal structure of USP7 binding with DNMT1 (615–1600) was determined, indicating that USP7 binds to the KG linker region, which might restrict the full-length DNMT1 to adopt certain conformations in solution [15]. In this study, the complexes of full-length DNMT1-H3Ub and DNMT1-USP7 were constructed by structural modeling (Figure 1D,E).

To date, multiple studies have demonstrated that DNMT1 mutations could lead to a spectrum of neurodegenerative disorders [16], such as hereditary sensory and autonomic neuropathy type 1E (HSAN1E) and autosomal dominant cerebellar ataxia, deafness, and narcolepsy (ADCA-DN) [17]. All of the disease-associated mutations are located in the RFTS domain of DNMT1. Since the RFTS domain is a hub for DNMT1 intra- and inter-molecular interactions, these mutations could potentially affect the localization, activation, and/or inhibition of DNMT1. On the other hand, DNMT1 undergoes several post-translational modifications (PTMs), including phosphorylation, methylation, ubiquitination, acetylation, and sumoylation [18]. Unlike disease mutations, the structural mapping shows that PTMs are located at the linker regions connecting the domains and on the surfaces of the DNMT1 domains, where they can directly affect the conformation of the enzyme and influence the interactions with other proteins. For example, phosphorylated Ser515 is involved in the interactions between the N-terminal and CD and is necessary for the activity of DNMT1 [19], whereas the phosphorylation of Ser127 and Ser143 regulates the interactions of DNMT1 with PCNA and UHRF1 [20]. Due to their structural distributions, the mutations and PTMs may display different influences on the allosteric regulation of DNMT1, which still needs to be elucidated.

Allostery is a key molecular mechanism underpinning control and modulation in a variety of cellular processes [21,22]. Classical allosteric regulations have focused on ligand binding, while recent studies show that allosteric regulations can be stimulated by the perturbation of other types of functional sites, including mutations or amino acid substitutions, and PTMs [23]. Moreover, the activity of DNMTs was found to be regulated in a concerted manner by protein-protein interactions and cellular pathways [24]. As Apo-DNMT1 plays its function by exploiting inter-domain and molecular interactions, or “cross-talk”, the functional mechanisms of DNMT1 complexes would present a more complex interactome landscape. The deep understanding of the molecular origin of allosteric mechanisms underlying mutations and PTMs in protein-protein interactions proposes a new paradigm for structure-based drug discovery for DNMT1 [25,26].

Emerging computational tools provide new insights into understanding protein dynamics and allosteric effects [27]. With their recent development, biophysical simulations have enabled numerous detailed investigations for conformational dynamic processes in large protein systems at atomic resolution, especially for the mechanistic role of disease-causing mutations [28] and PTM sites [29]. There are a few computational studies that focus on structural, dynamic, and allosteric aspects of DNA methyltransferases. The molecular design and binding selectivity of inhibitors targeting DNMT1 and DNMT3A have been investigated by molecular dynamics (MD) simulations [30]. Recently, we have used a combination of biophysics and bioinformatics techniques including elastic network models (ENM) for characterizing collective movements and associated hinge sites, perturbation response scanning (PRS) and dynamic network analysis for identifying key residues and pathways, as well as sequence theoretic analysis for detecting conservation and coevolution patterns involved in allosteric signals of DNMT3A [31] and DNMT1 [7]. Herein, we apply this integrated computational approach [32] to characterize the allosteric dynamics of DNMT1s at an increasing level of complexity-from monomer to different heterodimeric regulatory complexes. With focus on DNMT1-H3Ub/USP7 complexes, our study has proposed molecular signatures of activating mutations and PTMs, and revealed general mechanistic aspects of PPI-induced signaling in DNMT1 complexes.

## 2. Results

**Figure 2:**
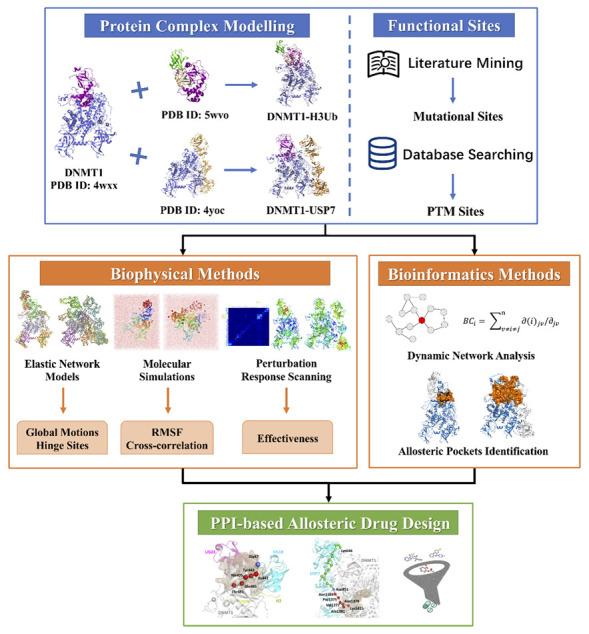
The computational workflow of using elastic network models, molecular dynamics simulations, structural residue perturbation, network modeling, and pocket pathway analysis to study allosteric dynamics and drug design in DNMT1-H3Ub/USP7 complexes.

In this work, we aim to evaluate differences in dynamics and allosteric interactions of Apo-DNMT1 and the two binding protein complexes (DNMT1-H3Ub and DNMT1-USP7), especially the intra- and inter-allosteric interactions landscape induced by interactors, mutations, and PTMs. We first compared the global motions and atomic-level correlations among three DNMT1 systems, followed by allosteric potentials and communication pathways as predicted by PRS and network analysis. We then discussed the possible allosteric mechanisms of the DNMT1-H3Ub and DNMT1-USP7 complexes, as well as their implications for future drug design. The computational framework of this work is illustrated in Figure 2.

### 2.1. Structural Data of DNMT1 Complexes

In DNMT1–H3Ub, the two ubiquitins *bind* to the N-lobe of RFTS through a combination of hydrophobic and hydrophilic interactions (Figure 1D). The core structure of the N-lobe in RFTS consists of six anti-parallel strands, β1-β6, and three helices, α1-α3. The α4 helix connects the N-lobe and C-lobe, with the latter half forming a four-helix bundle with α5-α7 in the C-lobe. Specifically, the loop structure consisting of residues 387–403 in the N-lobe is induced upon the binding of two ubiquitins, which was disordered in Apo-hDNMT1 and hence named as the ‘‘ubiquitin recognition loop (URL)’’. The H3 peptide is just located at the cleft between the two lobes. The binding of H3Ub contributes to a spatial rearrangement of the two lobes, leading to the opening of its catalytic site. However, the dynamics of DNMT1-H3Ub and the effect caused by binding with the H3-ubiquitin to DNMT1 remain a mystery to be answered.

In the DNMT1-USP7 structure, USP7 consists of three separate modules, UBL1–2, UBL3, and UBL4–5 (Figure 1B,E), which exhibit an extended conformation beside DNMT1. In the DNMT1-USP7 complex, USP7 binds to the BAH2 domain and TRD region of DNMT1 on two separate interfaces (Interface-1 and Interface-2). Interface-1 primarily involves the positively charged KG linker of DNMT1 (residues 1109–-1119) and an acidic groove in UBL1–2 of USP7. Although the inter-molecular interaction is considered to be required for USP7-mediated stabilization of DNMT1, the detailed allosteric regulation caused by USP7 still needs to be answered.

Through mapping to the DNMT1 structure, 13 mutations occur in the RFTS domain (Figure 1C), which are important for the enzymatic activity, function, and subcellular localization of DNMT1 [3]. Due to the essential role of the RFTS domain, some of these mutations were reported to affect the interaction of DNMT1 with DNA [33], or other proteins, i.e., UHRF1 [34], thus leading to dysfunction of DNMT1 and related diseases. In addition, 17 PTM sites have been identified (Figure 1C) from the PhosphoSitePlus database [35]. PTMs localize in the crucial parts of DNMT1: in the catalytic domain, where they might directly regulate enzymatic activity, on the surfaces of the DNMT1 domains, where they can regulate the interaction with other proteins, and in the linker regions connecting the domains, where they can allosterically regulate the conformation of the enzyme. The mutations and PTM sites are listed in Tables S1 and S2.

### 2.2. Modulation of Global Motions of DNMT1 upon Complex Binding

**Figure 3:**
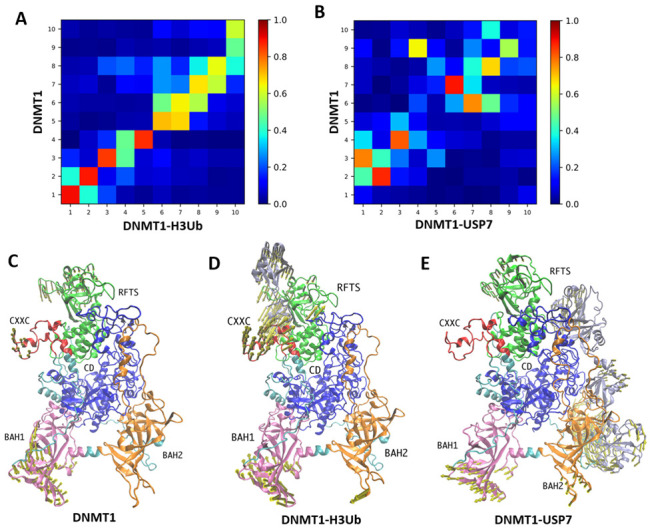
Intrinsic dynamics of three DNMT1 systems. (**A**) Overlaps between the ten slowest modes of DNMT1 monomer in isolated and H3Ub binding complex. (**B**) Overlaps between the ten slowest modes of DNMT1 monomer in isolated and USP7 binding complex states. The motions of the second ANM modes for the isolated (**C**) DNMT1, (**D**) DNMT1-H3Ub, and (**E**) DNMT1-USP7.

The intrinsic dynamics of allosteric proteins are defined by their topology of inter-residue contacts and favor cooperative motions (or global modes) that decide most of the functional mechanisms including the binding actions. To investigate how the complex binding modulates the cooperative motions of DNMT1, we first compare the ANM modes obtained for Apo-DNMT1 with those within the complexes, using the subsystem/environment coupling methods. Herein, the DNMT1 structure represents the subsystem, and the H3-ubiquitin and USP7 stand for the environment. The overlaps between the 10 lowest-frequency modes accessible to Apo-DNMT1 and within DNMT1-H3Ub/DNMT1-USP7 are displayed in Figure 3A,B, respectively. In both complexes, some global motions of Apo-DNMT1 are conserved to some extent. For DNMT1-H3Ub, the first three global modes of Apo-DNMT1 are basically conserved upon ubiquitinated H3 binding, in which the first mode shows the coupling motions between the RFTS and BAH2 domains (Figure S1A), the second mode shows the coupling motions between the RFTS and BAH1 domains (Figure 3C), while the third mode displays the collective motion of all corner domains of DNMT1, including RFTS, BAH1, and BAH2 (Figure S1B). However, in DNMT1-USP7, the overlap map shows a different pattern, in which the second mode is still conserved but the first and second modes are reordered to the 3rd and 4th modes upon USP7 binding. Thus, the environment provided by USP7 greatly affects the intrinsic dynamics of DNMT1, supporting the experimental results that the intermolecular interactions are required for USP7-mediated stabilization of DNMT1 [15].

The comparative examination among overlap maps indicates that the 2nd mode is well conserved in both complexes, indicating its importance in enzymatic regulation. This conservation may be caused by the small surface area for H3Ub binding and the larger but loosely protein-protein interface in the DNMT1-USP7 complex. For the 2nd mode in three DNMT1 systems, the conserved collective motion of Apo-DNMT1 shows the asymmetrical domain movement of RFTS and BAH1, which also highlights the important role of RFTS in DNMT1 regulation (Figure 3D,E). The motion direction of RFTS is changed in DNMT1-H3Ub, which may be caused by the rotation of the N-lobe upon the binding of ubiquitin. In two DNMT1 complexes, both of the 2nd modes display the large displacement of partner proteins, especially for the UBL1–2 module of USP7, which enjoyed the largest flexibility. Therefore, we can propose that such large-scale motions may be important carriers of allosteric signals for DNMT1 in all three states.

**Figure 4:**
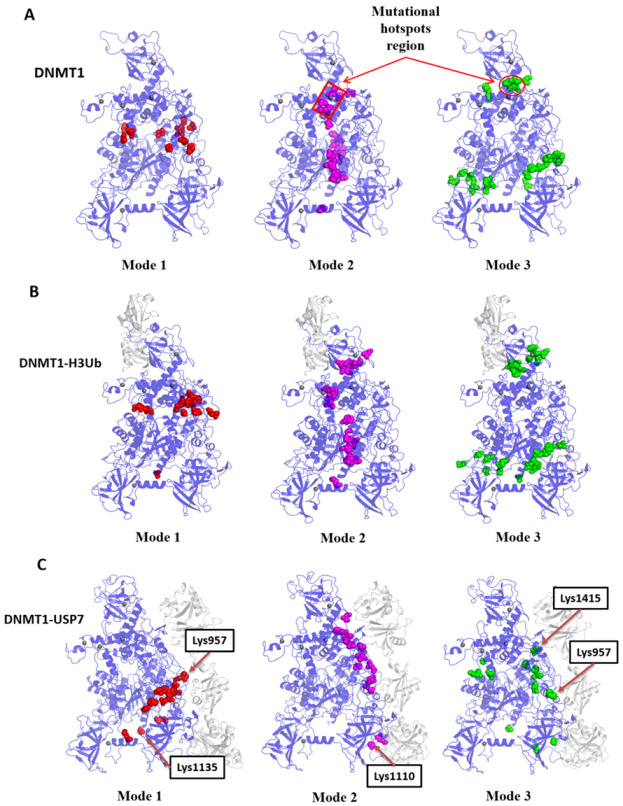
The prediction and structural mapping of hinge sites of (**A**) DNMT1, (**B**) DNMT1-H3Ub, and (**C**) DNMT1-USP7 based on their GNM modes 1, 2, and 3. The catching mutational and PTM sites are indicated by arrows.

In the collective dynamics, the hinge sites act as the pivot for coupling motions between domains, which were predicted by GNM mode calculation based on the local minima along their mode profiles. As shown in Figure 4A, the hinge sites of Apo-DNMT1 predicted by three lowest-frequency GNM modes are located at the RFTS-CD interface (mode 1), the vertical axis (mode 2), and triangle parts (mode 3) within the protein body. These distributions of hinge sites in DNMT1-H3Ub were just identical to the situations in Apo-DNMT1 (Figure 4B). The most interesting observation is that hinge regions predicted by modes 2 and 3 can catch certain disease mutations, including Lys505, Tyr524, Ile531, His553, Gly589, and Val590 in RFTS. This suggests that the mechanical changes of these mutations could be the potential driver for disease phenotypes. However, in DNMT1-USP7 (Figure 4C), the hinge sites corresponding to the three lowest-frequency GNM modes show different types of spatial distribution. In GNM mode 1, hinge regions include Met902, Val956, Pro988, Lys1135, Ser1358, Arg1363, Tyr1388, Phe1396, Gln1397, Gln1399, Gly1402, Pro1407, and Ala1600 in DNMT1, and Phe888 in USP7, indicating the coupling motions between RFTS-CXXC in DNMT1 and UBL1–2 in USP7. In GNM mode 2, the hinge sites are located in the interface of DNMT1 and USP7, including residues Asn541, Lys1110, Gln1123, Ser1372, Ser1394, Arg1401, Ile1428, Pro1440, Leu1455, Phe1492, Ile1496, Glu1543, and Ser1549 in DNMT1 and Pro556 in USP7. In GNM mode 3, the hinge sites are also located in the interface of RFTS and CD, including residues Gly614, Thr619, Asn701, Lys957, Lys981, Pro1106, Pro1127, Asn1415, Leu1517, Pro1530, Val1548, and Ala1600 in DNMT1 and Arg793 in USP7, just as in DNMT1 and DNMT1-H3Ub. The predicted hinge regions include some PTMs, including Lys957, Lys1110, and Lys1415 in DNMT1-USP7 just locating or close to the binding interface. The shifting of the hinge sites may present a functional mechanism, in which the different coupling dynamics introduced by USP7 binding are probably regulated by these PTM residues.

### 2.3. MD Simulations Reveal More Dynamic Plasticity in DNMT1 Complexes

To investigate the atomic interactions, 400-ns MD simulations were performed on three DNMT1 systems. As shown in Figure S2A, the global dynamical behavior of each simulated system was first characterized by root mean square deviations (RMSDs) values computed on the Cα atoms relative to the initial structure. This analysis evidenced that good RMSD convergences are observed for all three systems, although DNMT1-USP7 shows larger RMSDs (the red curve) and reaches equilibrium at 320 ns. By using data from MD simulations, we further explore the functional displacements using two fundamental and wide metrics: Root Mean Square Fluctuation (RMSF) and Dynamic Cross-Correlation Matrix (DCCM).

**Figure 5:**
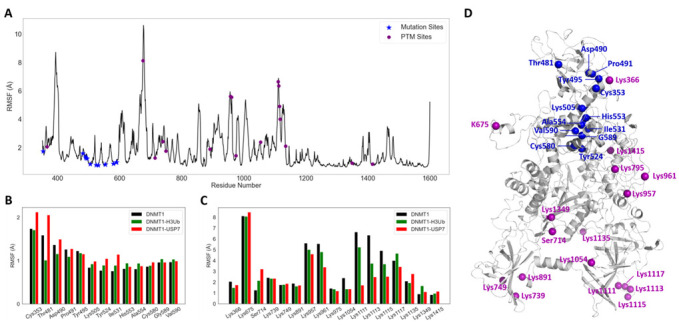
Residue fluctuations of three DNMT1 systems. (**A**) RMSF values per residue based on the MD simulation of Apo-DNMT1. RMSF values for each (**B**) disease mutation and (**C**) PTM in three DNMT1 systems. (**D**) Key mutations and PTMs are indicated on the DNMT1 structure.

RMSF values per residue along the MD simulation of Apo-DNMT1 are shown in Figure 5A, suggesting peaks were observed at several prominent loops, with the two highest values in the ubiquitin recognition loop RFTS and the loop in CXXC. By mapping disease mutations and reported PTMs on this RMSF profile, we can observe that all disease mutations display highly stabilities, whereas PTMs enjoy a significant flexibility, including Lys675 at the loop in CXXC, Lys957 and Lys961 at the loop in BAH2, and Lys1111, Lys1113, Lys1115, and Lys1117 at the loops in interface-1. In comparison with the isolated state, the H3Ub binding (Figure S2B) will of course stabilize the interface region (residues 351–410), especially the binding loop at RFTS, and increase the fluctuation of the TRD region (residues 1340–1360) between RFTS and CD. In DNMT1-USP7, the larger binding interface including four acetylation sites becomes stable, and some regions far away from the interface become more flexible, including the CD, residues 370–385 in RFTS, and residues 830–850 in BAH1. In addition, the changes in fluctuations of disease mutations and PTMs in three DNMT1 systems have been systematically compared. Overall, disease mutations in DNMT1-USP7 and PTMs in isolated DNMT1 show larger fluctuations. Importantly, we have found that three mutation sites Thr481, Asp490, and Pro491 become more stable upon H3Ub binding (Figure 5B), and three interfacial PTM sites show considerable conformational fluctuation in DNMT1-USP7 (Figure 5C). The change in flexibility upon protein-protein interactions not only happens at the binding regions but also in the mutational and PTM sites (Figure 5D). We hypothesize that these differences in conformational fluctuations upon these three events potentially contribute to the allosteric change.

**Figure 6:**
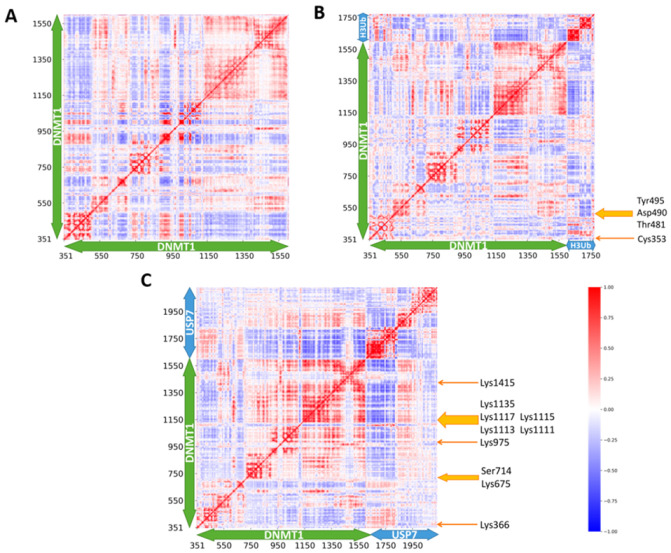
The cross-correlation maps in MD simulations of (**A**) DNMT1, (**B**) DNMT1-H3Ub, and (**C**) DNMT1-USP7. Positive values indicate residues that displace in the same direction, whereas negative values are associated with the opposite displacement, as color gradient of red and blue, respectively.

We further evaluated the intra- and inter-molecular cross-correlations based on the MD simulations between residue fluctuations with the consideration of atomic interactions. It is shared in different DNMT1 systems that the coupled regions are in line with the modular architecture and quaternary arrangement. In Apo-DNMT1 (Figure 6A), strong intra-domain correlations within the RFTS and CD domains were observed, which behave as rigid bodies. In addition, some weak intra-domain correlation within the BAH1 and BAH2 domains and relative inter-domain correlations between the RFTS, CD, and BAH domains were also found. Our previous work based on normal mode analysis showed a similar result, suggesting both intra- and inter-domain correlations [7]. As similar intrinsic dynamics are observed in Apo-DNMT1 and DNMT1-H3Ub (Figure 6B), the H3-ubiquitin binding has very limited effects on the cross-correlations within the DNMT1 residues, and only weakly enhances the intra-domain correlation between two lobes of the RFTS domain. In the coupling motions between DNMT1 and H3-ubiquitin, the ubiquitins have positive coupling motions with the N-lobe of the RFTS and BAH2 domains, especially for four disease mutations of Cys353, Thr481, Asp490, and Tyr495. However, in DNMT1-USP7 (Figure 6C), the residues in BAH2 have strengthened positive coupling correlations with the CD compared with DNMT1. On the other hand, the positive correlations suggest inter-molecular interactions between USP7 and all domains in DNMT1, even with the BAH1 domain. Most PTM sites including Lys366, Lys675, Ser714, Lys975, Lys1111, Lys1113, Lys1115, Lys1117, Lys1135, and Lys1415 are involved in the coupling motions with USP7.

The conformational dynamics results showed the promiscuity of functional regulation in the protein complex of DNMT1, which was not only decided by the intra-molecular interactions but also the inter-molecular interactions with H3Ub/USP7, as well as functional cross-talk between mutational and PTM sites.

### 2.4. DNMT1–H3Ub/USP7 Are High-Potential Signaling Complexes

In this section, perturbation response scanning (PRS) and network-based approaches were applied to quantify the allosteric potential of two DNMT1 complexes: DNMT1-H3Ub and DNMT1-USP7. Based on ANM calculation, PRS analysis was employed to quantify the allosteric effect of each residue in the DNMT1 complex structures on all other residues in response to external perturbations. Regarding the PRS map, with the information on the sensitivity and effect of certain residues in transmitting signals, two groups including sensors and effectors were predicted to be potentially involved in allosteric signal sensing and transmission. As shown in Figure S3, the primary results of PRS maps for three systems including Apo-DNMT1 show that two DNMT1 complexes have higher allosteric potential. In general, Apo-DNMT1 has higher intra-allosteric properties by H3Ub binding, especially for the RFTS domain, whereas its intra-monomer allosteric ability looks similar to DNMT1-USP7. The high allosteric potential of H3UB and USP7 suggests relatively strong inter-protein allosteric interactions in both DNMT1 complexes, with the highest allosteric potential found for UBL1–2 of USP7.

**Figure 7:**
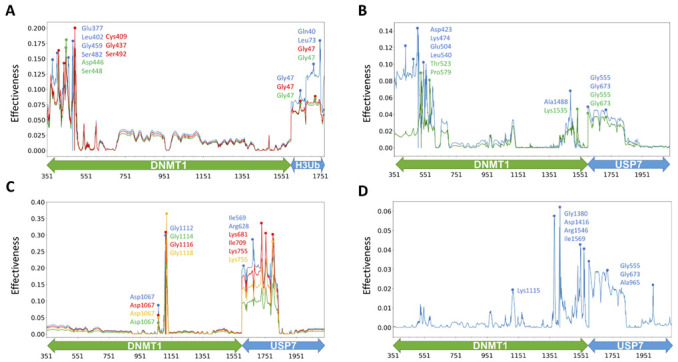
PRS results for some disease mutations and PTMs in two DNMT1 complexes. Effectiveness profiles for (**A**) DNMT1-H3Ub by the perturbation of three disease mutational hotspots of Thr481 (the blue line), Asp490 (the green line), and Pro491 (the red line), for DNMT1-USP7 by the perturbation of (**B**) two disease mutational hotspots of Lys505 with the blue line, Tyr524 with the green line, (**C**) four interfacial acetylation sites (Lys1111 with the blue line, Lys1113 with the green line, Lys 1115 with the red line, Lys1117 with the orange line) and (**D**) Lys 1415 at distant site.

**Figure 8:**
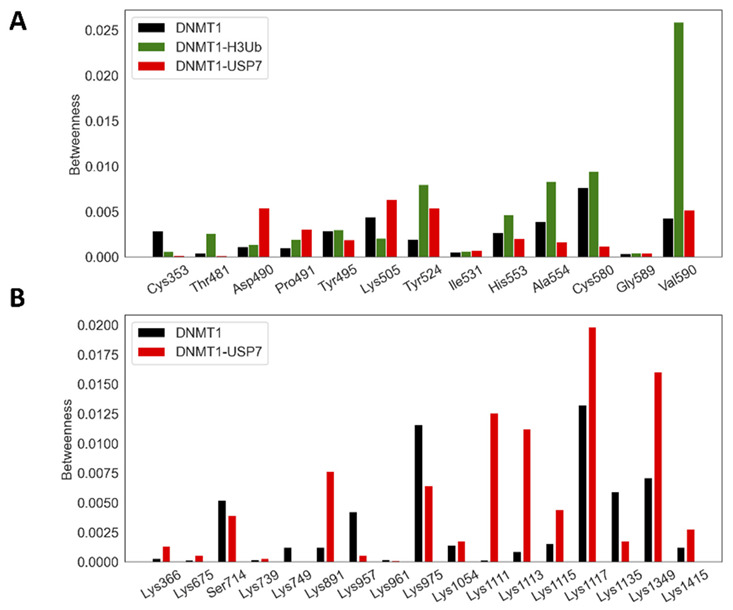
The comparison of BC values for (**A**) each disease mutation in three DNMT1 systems and (**B**) PTM sites in DNMT1 and DNMT1-USP7.

Meanwhile, network analysis based on MD simulation, so-called dynamic network analysis, would give more dimensions of how residues involved in the signal transmit in terms of some network parameters. Our studies on other protein systems have proved that PRS effectiveness [36] and network betweenness centrality (BC) [37] are particularly useful to quantify molecular signatures of single sites and their abilities in signaling transmission. Therefore, herein, we also employed these two matrices to account for the allosteric dynamics of DNMT1 complexes. The systems analysis of the perturbation effects for mutational hotspots and PTMs was first performed by the calculation of PRS effectiveness. In DNMT1-H3Ub, only three mutational hotspot sites (Thr481, Asp490, and Pro491) were found to have similar strong perturbation effects (Figure 7A), which include local interactions with Glu377, Glu400, Leu402, Phe434, Gly459, Asn462, Phe479, Ser480, Ser482, and Phe483 within the RFTS domain, as well as long-range allosteric interactions with Gln40, Gly47, and Leu73 located at H3Ub. The signaling importance of these mutations was also predicted by the higher values of betweenness in DNMT1-H3Ub compared with the isolated monomer (Figure 8A). In addition, five other mutations including Tyr524, His553, Als554, Gly589, and Val590 also showed larger betweenness, which may be caused by hydrogen bond interactions between RFTS–CD domains to enhance their local interactions and inter-domain signal transmission. In DNMT1-USP7, two mutational hotspots (Lys505 and Tyr524) and four interfacial acetylation sites (Lys1111, Lys1113, Lys 1115, and Lys1117) were caught by higher values of effectiveness and betweenness centrality. The perturbations of Lys505 and Tyr524 show similar allosteric effects, and while they have long-range interactions with Gly555, Gly673 in USP7 may go through some local interactions with Asp423, Lys474, Glu504, Leu540, Thr523, and Pro579 within the RFTS domain (Figure 7B). Two additional mutations (Asp490 and Pro491) show much higher betweenness centrality (Figure 8A), increasing the signaling transformation between the RFTS and CD domains. The inter-molecular communication of four acetylation sites (especially Lys1111 and Lys1113 with the highest betweenness centrality changes, Figure 8B) was highlighted here to have long-range interactions with the UBL1–2 domain (Ile569, Arg628, Asp682, Ile709, and Lys755) of USP7 (Figure 7C). Expect for these interfacial PTMs, the perturbation of PTM Lys1415 at the distant site also shows inter-molecular and long-range allosteric communications with UBL1–2 (Gly555, Gly673, and Ala965) in USP7 (Figure 7D). The increasing betweenness centrality of PTM sites (Lys891 and Lys1349) may correspond to some stronger intra- and short interactions.

In summary, PRS and network analysis found that some particular mutational and PTM sites serve as “hub sensing”, and their induced effects contribute to local and long-distance structural rearrangements interpreted as allosteric events. The inter-protein allosteric pathways originating at mutations and PTM residues are particularly important to understand the regulatory mechanisms of protein complexes, which need to be further established.

### 2.5. Allosteric Communication Pathways in DNMT1-H3Ub/USP7

The above results suggest three scenarios of inter-monomer allosteric interactions as follows: (1) mutational hotspots Thr481, Asp490, and Pro491, associated with HSAN1E, have long-range effects on H3Ub; (2) mutational hotspots Lys505 and Tyr524 show long-range effects on USP7; and (3) PTM sites Lys1111, Lys1113, Lys1115, Lys1117, and Lys1415 have long-range effects on USP7. Using dynamic network data, we further analyzed allosteric pathways originating at these allosteric mutations and PTMs in DNMT1 and terminating at the most perturbation sites at the partner protein. The detailed analysis of all the pathways listed in Table S3 would add a new insight to the understanding of the crucial role of intrinsic molecular dynamics in mediating protein–protein signaling in DNMT1 complexes.

**Figure 9:**
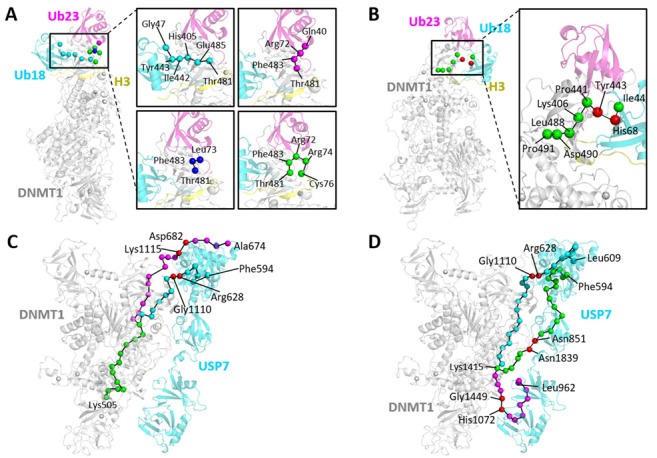
Allosteric communication pathways in DNMT1-H3Ub and DNMT1-USP7. (**A**) Four important pathways (indicated by cyan, magenta, blue, and green spheres) with Thr481 in DNMT1 as the source and Gly47 located at Ub18 and Gln40, Leu73, and Cys76 at chain Ub23 in H3UB as targets. (**B**) A pathway pattern by using Asp490 and Pro491 as starting points. (**C**) Two important pathway patterns (colored in cyan and magenta) starting from Lys505 in DNMT1–USP7 complex. They have the same path on DNMT1, which is colored green. (**D**) Three path patterns (colored in cyan, magenta, and green) induced by the acetylation site of Lys1415 in USP7. The important residue pairs that transmit signals at the protein interface are expressed by red spheres.

In DNMT1-H3Ub, two types of communication pathways were observed, one with Thr481 as the source residue, and the other with Asp490 and Pro491 as the source residues. It is shared that both types of pathways have identical terminuses in ubiquitins. Starting from Thr481 (Figure 9A), four important unique pathways are (DNMT1) Thr481→Glu485→His405→Ile442→Tyr443→ (H3Ub. Ub18) Gly47, (DNMT1) Thr481→Phe483→ (H3Ub. Ub23) Arg72→Gln40, (DNMT1) Thr481→Phe483→ (H3Ub. Ub23) Leu73, and (DNMT1) Thr481→Phe483→ (H3Ub. Ub23) Arg72→Arg74→Cys76. Starting from Asp490 and Pro491 (as an example in Figure 9B), the most possible pathway patterns are (DNMT1) Pro491→Asp490→Leu488→Lys406→Pro441→Tyr443→ (H3Ub. Ub18) His68→ (H3Ub. Ub18) Ile44, (DNMT1) Pro491→Asp490→Leu488→Lys406→Pro441→Tyr443→ (H3Ub. Ub18) Gly47, (DNMT1) Pro491→Asp490→Leu488→Leu476→ (H3Ub. Ub18) Thr9→Thr7→Val70, and (DNMT1) Pro491→Asp490→Leu488→Tyr486→Gln404→Leu402→ (H3Ub. Ub23) Thr9→Leu8. Through pathway analysis, some key residue pairs involved in inter-molecular communications are (DNMT1) Tyr443–(H3Ub. Ub18) Arg47 and (DNMT1) Ser482/Phe483–(H3Ub. Ub23) Arg72/Leu73, which participate in pathways starting from all three mutations, as well as (DNMT1) Tyr443–(H3Ub. Ub18) His68, which is the most frequent residue pair among all pathways.

In DNMT1-USP7, starting with mutational hotspot Lys505, two types of communication pathways with quite a long length were found (Figure 9C). The first type is also the major type using (DNMT1) Gly1110-(USP7) Arg628 as the bridge to transfer information through DNMT1 to USP7, such as (DNMT1) Lys505→His553→Ser549→Phe545→Ile531→Leu527→Tyr524→Gly1534→Gln1536→Ser1524→Phe1522→Thr1366→Thr1364→Phe1362→Ile1039→Asn1040→Tyr1084→Tyr1064→Asp1067→Asn1108→Gly1110→ (USP7) Arg628→Gln626→Pro624→Thr659→Val567→Phe594. The other type uses (DNMT1) Lys1115-(USP7) Asp 682 as another bridge to transfer inter-molecular communications, such as (DNMT1) Lys505→His553→Ser549→Phe545→Ile531→Leu527→Tyr524→Gly1534→Gln1536→Ser1524→Phe1522→Thr1366→Thr1364→Val1333→Pro990→Arg992→Gly994→Ile996→Val935→Gln1123→Lys1121→Lys1119→Lys1117→Lys1115→ (USP7) Asp682→Asp680→Lys678→Leu676→Ala674. Given the starting point at Tyr524, two similar types of pathways were predicted. One uses (DNMT1) Gly1110-(USP7) Arg628 as the bridge, including (DNMT1) Tyr524→Gly1534→Gln1536→Ser1524→Phe1522→Thr1366→Thr1364→Phe1362→Ile1039→Asn1040→Tyr1084→Tyr1064→Asp1067→Asn1108→Gly1110→ (USP7) Arg628→Gln626→Pro624→Thr659→Val567→Phe594. The other one uses (DNMT1) Lys1115→ (USP7) Asp682 as the mediator, such as (DNMT1) Tyr524→Gly1534→Gln1536→Ser1524→Phe1522→Thr1366→Thr1364→Val1333→Pro990→Arg992→Gly994→Ile996→Val935→Gln1123→Lys1121→Lys1119→Lys1117→Lys1115→ (USP7) Asp682→Asp680→Lys678→Leu676→Ala674. It should be noted that these two types of pathways both suggested the importance of interfacial PTMs (Lys1115) or their neighbors (Gly1110) in protein–protein interaction-based allosteric communications.

However, for PTM-induced pathways in DNMT1-USP7, four interfacial PTMs show much smaller lengths but are more promiscuous. For example, although Lys1111 always forms an interaction with Glu759 in USP for the allosteric transformation, it can also interact with residues Arg628, Ser629, and Asn630 located at the USP7 UBL1–2 domains. For Lys1113, the most important mediator is (DNMT1) Lys1113-(USP7) Glu759. However, for Lys1115, the most connecting residues not only include Glu759 but also Leu760, which means four import residue pairs (DNMT1) Lys1115-(USP7) Glu759, (DNMT1) Lys1115-(USP7) Leu760, (DNMT1) Lys1117-(USP7) Glu759, and (DNMT1) Lys1117-(USP7) Leu760. The most interesting PTM is Lys1415, which generates some long-range allosteric pathways, while some representative examples (Figure 9D) are (DNMT1)Lys1415→Asn1379→Arg1378→Glu1376→Pro1375→Asn1389→(USP7)Asn851→Lys841→Leu876→Val796→Thr812→Arg793→Tyr791→Pro740→Asn581→Asp582→Lys633→Met625→Ile660→Gln568→Phe594, (DNMT1)Lys1415→Cys1414→Val1548→Glu1552→Met1371→Asp1369→Tyr1035→Ala1037→Leu1041→Tyr1084→Tyr1064→Arg1104→Ser1105→Asn1108→Gly1110→(USP7)Arg628→Gln626→Pro624→Leu622→Ile620→Gln617→Val606→Leu609, and (DNMT1)Lys1415→Met1417→Ala1419→Leu1446→Gly1449→(USP7)His1072→Arg1074→Trp1076→Thr991→Ile949→Leu942→Phe970→Pro964→Leu962, which include three key inter-molecular bridges such as (DNMT1)Asn1389-(USP7) Asn851, (DNMT1)Gly1110-(USP7)Arg628, and (DNMT1)Gly1449-(USP7)His1072. Thus, Lys1415 was suggested as a critical acetylation site for the integration of long-range allosteric signals.

### 2.6. Allosteric Pockets Regulate DNMT1 Complexes through Protein–Protein Interactions

In addition to allosteric communication pathways, the detection and characterization of binding pockets in proteins are also crucial for studying biological regulation and drug design. Based on MD simulations, Allosite [38] and AlloPred [39] were used to detect the allosteric pockets in DNMT1 protein complexes. Overall, 21 and 8 potential allosteric pockets were identified in DNMT1–H3Ub and DNMT1–USP7, respectively. The allosteric binding properties including volume, total solvent-accessible surface area, feature score, perturbation score, AlloSite, and druggabilty scores are listed in Tables S4 and S5.

**Figure 10:**
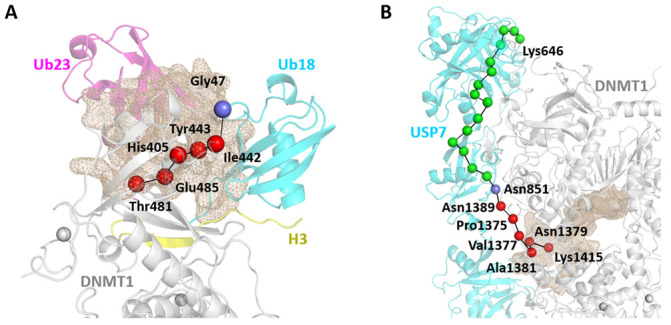
Allosteric pockets and the corresponding communication pathways detected in DNMT1 complexes. (**A**) Pocket 4 in DNMT1-H3Ub and the involved pathway induced by the mutational hotspot Thr481. (**B**) Pocket 3 in DNMT1-USP7 and the involved pathway induced by the PTM site Lys1415. The pockets are shown as wheat contoured meshes. The residues of each pathway are represented by green spheres, where common pathway patterns are highlighted in red (DNMT1) and slate (H3UB.Ub18/USP7).

In Apo-DNMT1, we studied pocket 4 [7], which is located at the RFTS-CD domain interface and acts as a potential allo-targeting site. This allosteric pocket was largely conserved in DNMT1-H3Ub, which was detected as pocket 1 in this protein complex (Figure S4A). This pocket contains some mutational hotspot residues (Lys505, His553, Ala554, Gly589, and Val590), involving crucial hinge residues in the GNM collective motions (Lys366, His418, Asp423, Leu426, and Ile498). Moreover, it takes part in the local allosteric networks in the C-lobe of the RFTS and TRD regions, but was not captured in the inter-protein communication pathways. In addition, two allosteric pockets were found located at the DNMT1-H3Ub interface, namely pockets 4 and 5, with druggability scores of 0.465 and 0.065. Pocket 4 is located at the interface consisting of Ub23 in H3Ub (Figure 10A), including Thr481 whose mutation to Pro would cause HSAN1E. Many residues in this pocket are involved in long-range allosteric pathways through the DNMT1-H3Ub interface. For example, four pocket residues Thr481, Glu485, His405, and Ile442 are involved in the pathway induced by the mutational hotspot Thr481, that is ((DNMT1) Thr481→Glu485→His405→Ile442→Tyr443→ (H3Ub.Ub23) Gly47). Pocket 5 is located at the interface consisting of Ub18 in H3Ub (Figure S4B), and its consisting residues were also captured in some long-range paths for inter-protein communications induced by mutational hotspots of Thr481 and Asp490. By comparing these two interfacial pockets, we suggest that pocket 4 may be more interesting because it contains disease-causing mutational residues and it has reasonable druggability scores.

In DNMT1-USP7, fewer allosteric pockets have been predicted, although it binds to a larger protein partner. Pocket 1, pocket 2, and pocket 3 located at the DNMT1-USP7 interface are also of particular interest. Pocket 1 has the largest volume, consisting for the most part of the RFTS, BAH2, and CD domains in DNMT1 and the UBL4–5 domain in USP7 (Figure S5A). Due to its large size, its consisting residues can be captured by experimental and computational results: (1) mutational hotspots (Tyr495, Lys505, Ile531, His553, Gly589, and Val590), associated with two types of disease phenotypes; (2) some hinge sites (Leu542, Asp1416, and Leu1455) in collective motions; (3) and long-range communications through protein–protein interactions induced by Lys505 and Tyr524. Pocket 2 also displays relatively large size, located at interface-2 and circumscribed by tails of the BAH2 and CD domains in DNMT1 and UBL3 in USP7 (Figure S5B). The pocket residues can be captured by more hinge sites, which means that this pocket plays important roles in global motions by connecting DNMT1 and USP7 to behavior like a rigid body. Although this pocket does not include any mutational hotspot residues, it is also largely involved in inter-protein commutations, which may be induced not only by Lys505 and Tyr524, but due to the most collective motion along the interface. In comparison with these two main large pockets, pocket 3 has the smallest size but the largest druggability score of 0.375 (Figure 10B). Structurally, this pocket is located at the DNMT1-UBL4 interface and includes the RFTS and CD domains in DNMT1 and the UBL4 domain in USP7. Among pocket residues, we have paid attention to the acetylation site Lys1415, whose perturbation pathway to USP7, such as (DNMT1) Lys1415→Asn1379→Ala1381→Val1377→Pro1375→Asn1389→ (USP7) Asn851, may be stimulated by targeting this pocket.

Pocket pathway analysis suggests two potential allosteric pockets, pocket 4 in DNMT1-H3Ub and pocket 3 in DNMT1-USP7, which can regulate the allosteric pathways in DNMT1 complexes induced by disease-causing mutations and PTMs through protein-protein interactions.

## 3. Discussion

DNA methylation, as a major epigenetic modification, plays an important role in the regulation of transcription, genome stability, genomic imprinting, X-chromosome inactivation, and retrotransposon silencing. In mammals, DNA methylation mainly occurs on the C5 position of cytosines within CpG dinucleotides. Mammalian DNA methylation is introduced by three active DNMTs including DNMT1. Structural and biochemical studies of DNMT1 have shown that its catalytic activity is under the allosteric control of N-terminal domains, including RFTS and CXXC, with autoinhibitory function [24]. On the other hand, DNMT1 always interacts with some partner proteins to maintain global DNA methylation during somatic cell divisions. The DNMT complexes were proposed to have novel regulatory principles, but the underlying mechanism is not well understood. Here, based on structural modeling of full-length DNMT1 protein complexes, we have elucidated the molecular basis of the long-range allosteric activation of DNMT1-H3Ub and DNMT1-USP7. In summary, we will discuss our work from the following three perspectives.

In our strategy, we preliminarily established a complementary computational framework to study allosteric mechanisms of DNMT1-H3Ub/USP7 complexes by integrating computer modeling, biophysical simulations, and bioinformatics methods. Through computational modeling, full-length DNMT1 in complex with interacting proteins was constructed, raising the possibility to investigate their complicated allosteric control effects. The post-genomic era witnesses the development of functional sites, including disease-related mutations and PTMs. Driven by these data including structures, mutations, and PTMs, biophysical and bioinformatics-based methods were used to dig these big data. Specifically, at the biophysical level, we used MD simulation to sample the conformational dynamics data, ENM for detecting global motions and the determined hinge sites, and PRS for allosteric perturbation analysis. At the bioinformatics level, the biological network of the complex was constructed, and the network parameters were used for quantitative research, so as to characterize the characteristics of mutant and PTM residues. Then, pocket pathway analysis based on support vector machine (SVM) established the connection between communication pathways and allosteric drug design.

On the allosteric mechanism study of DNMT1, our work has two research paradigm transformations, that is, from “multiple-domain monomer allostery” to “inter-molecular allostery” and from “conformational change based allostery’’ to “mutation and PTM-induced allostery”. Protein complexes favor functional motions involved in protein–protein interactions by enhancing the inter-molecular allosteric communications. It is broadly established that the allosteric regulations and biological functions of protein complexes are decided by their oligomerization states, global dynamics, and intermolecular interactions [40,41]. On the other hand, both mutational and PTM sites represent other important regulatory instruments that modulate the structure, dynamics, and function of proteins. Systems analysis of their molecular signatures and allosteric properties provides new insights into the origin and effects induced by these functional sites. In our work, for two protein complexes of DNMT1-H3Ub and DNMT1-USP7, we have given a landscape of allosteric pathways: originating at the mutational hotspots or PTM sites, through the allosteric protein–protein interface, and ending at the partner protein to control the cell activity.

The implication of our study in drug design is finally discussed. The reported mutations of DNMT1 are tightly associated with various diseases such as HSAN1E and ADCA-DN, potentiating them as important therapeutic targets. To this aim, DNMT1 inhibitors are mainly developed for targeting its catalytic pocket, but suffer from poor selectivity, weak activity, and toxic side-effects [26]. Targeting allosteric sites at the protein–protein interface of DNMT1 complexes may present an alternative strategy to develop epigenetic drugs with enhanced pharmacological profiles and druggability. Therefore, the general understanding of allostery based on protein–protein interactions in DNMT1-H3Ub/USP7 could be promising in guiding the development of such allosteric inhibitors in the future. Three interfacial pockets, localized in close proximity to mutational hotspot sites and involved in long-range communication pathways across DNMT1-H3Ub and DNMT1-USP7 interfaces, may constitute valid targets to develop inhibitors able to modulate the function-related communication properties of these two DNMT1 protein complexes.

## 4. Materials and Methods

### 4.1. Structural Models and MD Simulation

The crystal structure of human DNMT1 (351–1600) was selected for further analysis (PDB ID: 4WXX). The DNMT1-–USP7 complex was constructed by combining the two crystal structures: human DNMT1 (615–1600) in complex with USP7 (PDB ID: 4YOC) and the RFTS domain (351–614) of hDNMT1 (PDB ID: 4WXX). The structure of DNMT1-ubiquitinated H3 was constructed as follows: the crystal structure of hDNMT1 RFTS domain in complex with mono-ubiquitylated histone H3 (PDB ID: 5WVO) was superimposed to hDNMT1 by the N-lobe, then the ubiquitinated H3 was extracted from the RFTS-ubiquitinated H3 complex and combined with hDNMT1 (351–1600), leading to the DNMT1–ubiquitinated H3 complex. All the missing residues were added using Modeller and then the models were minimized by the Protein Preparation Wizard Workflow provided in the Maestro.

MD simulations were carried out by Amber14 package using ff14SB forcefield [42,43]. Protonation states of each system were predicted by H++. The zinc-bound sites were treated with Amber ZAFF parameters. The tleap module was utilized to record the topology of each system, then Na^+^ and Cl^−^ ions were added to neutralize the simulation systems [44]. Then, each system was solvated in the TIP3P [45] water with a buffer distance of 10 Å. Langevin thermostat was used to maintain pressure and temperature during the simulations [46]. Afterwards, each system was minimized in two phases. First, energy minimization was carried out with a restraint of 10 kcal/mol/Å on solute atoms (executing 500 steps of the steepest descent method followed by 500 steps of the conjugate gradient method). The steps in the second phase were the same as in the first phase, but no restrictions were imposed on any molecules. The cutoff for non-bonded interaction was set as 9 Å. Subsequently, each system was heated from 0 to 300 K in 100 ps with a time step of 2 fs and harmonic restraint of 10 kcal/mol/Å. After heating, equilibration of 100 ps with a restraint of 1 kcal/mol/Å was performed, following by another equilibration of 1000 ps with time step of 2 fs with no restrictions. Finally, a production run of 400 ns for each system was performed using pmemd.cuda with a time step of 2 fs, and the trajectories were saved after every 5000 steps.

### 4.2. Residue Fluctuations and Correlations Analysis

The resultant MD trajectories of each protein were analyzed in terms of residue root mean square fluctuations (RMSF), dynamic cross-correlation map (DCCM), and hydrogen-bond (H-bond) occupancy by the CPPTRAJ [47] module in AmberTools 18. RMSF per residue was calculated to identify flexible residues and to compare differences in dynamics among three DNMT1 systems. The calculation of DCCM is based on the diagonalization of 3N×3N covariance matrixes from the MD trajectory, whose elements are defined as:

(1) $$C_{ij}=\frac{\langle \left(R_{i}-\langle R_{i}\rangle \right)\left(R_{j}-\langle R_{j}\rangle \right)\rangle }{\sqrt{\langle R_{i}-\langle R_{i}\rangle \rangle {}^{2}\langle R_{j}-\langle R_{j}\rangle \rangle {}^{2}}}$$

where $R_{i}$ and $R_{j}$ denote the position of Ca atoms and the brackets denote the average over trajectory frames. For completely correlated motions, $C_{ij}=1$, and for completely anticorrelated motions, $C_{ij}=-1$. There is no correlation when $C_{ij}=0$.

### 4.3. Elastic Network Models

The anisotropic network model (ANM) and the Gaussian network model (GNM) are two kinds of elastic network models (ENMs) for performing normal mode analysis on low-frequency modes of proteins and their complexes. In ANM [48], each amino acid residue is represented by a node placed at its Cα coordinate. Residue pairs that fall within a cutoff distance rc = 15 Å are connected by harmonic springs of uniform force constant γ. For a network composed of N nodes, the total potential energy is a summation over all springs in the system and the (3N × 3N) Hessian matrix H is constructed based on the minimum energy structures, which is the same as the crystal structures. Diagonalization of H yields (3N-6) nonzero eigenvalues ($\lambda_{k}$) with corresponding eigenvectors ($u_{k}$). Starting with the first non-zero eigenvalue, $\lambda_{k}$ are sorted in ascending order, and also the corresponding $u_{k}$. The similarity between the ANM eigenvector sets described by two different states/conformations of a system can be calculated according to the following equation:

(2) $$Overlap\left(k\right)={\left(\frac{1}{k}\overset{k}{\underset{i=1}{\sum }}\overset{k}{\underset{j=1}{\sum }}{\left(u_{i}.v_{j}\right)}^{2}\right)}^{1/2}$$

Here, the inner product ($u_{i}.v_{j})$ quantifies the individual overlap between the *i*th and *j*th eigenvectors belonging to the different sets. Overlap (*k*) quantifies the overall correspondence between the first *k* modes of the sets. In GNM [49], instead of H, the (N × N) Kirchhoff/connectivity matrix is constructed from the structural coordinates with a cutoff distance of 10 Å. Eigenvalue decomposition yields (N-1) non-zero modes for a folded structure. In our work, the GNM is used for evaluating the residue mean-square fluctuations of proteins with their minimal values for predicting hinges. Both GNM and ANM analyses were performed by using the ProDy package [50].

### 4.4. Perturbation Response Scanning

The perturbation response scanning (PRS) approach is based on the linear response theory and allows the evaluation of residue displacements in response to external forces [51]. The PRS technique was combined with protein dynamics based on cross-correlations calculated from ANM by constructing the Hessian matrix H. The 3N-dimensional vector ΔR of node displacements in response to the application of a perturbation (a 3N-dimensional force vector F) obeys Hooke’s law F=H·ΔR. The idea in PRS is to exert a force of a given magnitude on the network, one residue at a time, and observe the response of the overall network. The force exerted on residue i is expressed as:

(3) $$F^{\left(i\right)}={\left(0\;0\;0\;\wedge F_{x}^{\left(i\right)}F_{x}^{\left(i\right)}F_{x}^{\left(i\right)}\wedge 0\;0\;0\right)}^{T}$$

The resulting response is:

(4) $$\Delta R^{\left(i\right)}=H^{-1}F^{\left(i\right)}$$

ΔR^(i)^ is a 3N-dimensional vector that describes the deformation of all the residues (in N blocks of dimension 3, each) in response to $F^{\left(i\right)}$. A metric for the response of residue k is the magnitude $\langle \|\Delta R_{k}^{\left(i\right)}{\|}^{2}\rangle$ of the kth block of ΔR^(i)^ averaged over multiple F^(i)^, expressed as the ikth element of the N × N PRS matrix, SPRS. The elements of SPRS refer to unit (or uniform) perturbing force. The response to unit deformation at each perturbation site is obtained by dividing each row by its diagonal value.

(5) $${\bar{S}}_{\mathrm{PRS}}=\left(\begin{array}{ccc} 1/d_{1} & & 0\\ & \ddots & \\ 0 & & 1/d_{N} \end{array}\right)S_{\mathrm{PRS}}$$

The average effect of the perturbed effector site i on all other residues is computed by averaging over all sensor (receiver) residues j and can be expressed as $\langle {\left(\Delta R^{i}\right)}^{2}\rangle {}_{\mathrm{sensor}}$. The effector profile $\langle {\left(\Delta R^{i}\right)}^{2}\rangle {}_{\mathrm{effector}}$ describes the average effect that local perturbation in the effector site i has on all other residues. The maxima along the effector and sensor profiles would correspond to functional mobile residues that undergo allosteric structural change.

### 4.5. Dynamic Network Analysis and Allosteric Pocket Identification

Dynamic network analysis was performed by the program of MD-TASK [52]. For each protein system, the network was constructed by extracting Cα and Cβ atoms of MD trajectories as nodes, and the edge is created when two nodes are within 6.5 Å. Then, betweenness centrality (BC) and shortest pathway were calculated based on the constructed dynamic network. BC is defined as the number of shortest paths running through a node/residue for a given network and provides a measure of usage frequency for each node during navigation of the network [53]. The communication pathways were calculated by pathways connecting “source” and “sink” residues with the shortest lengths (edges), using the Floyd-Warshall algorithm [54]. For allosteric pocket identification, two web servers of Allosite [38] and AlloPred [39] were performed by using average structures of DNMT1s over the MD simulation as input. Both methods combine the Fpocket algorithm and SVM classifier algorithms for cavity detection and pocket-based analysis, including their topological, physiochemical, and druggability features.

## 5. Conclusions

Taken together, our work has extended the research content of allosteric mechanisms from Apo-DNMT1 to DNMT1-H3Ub/USP7 complexes, that is, from allosteric signal transduction within a single protein to allosteric effects mediated by protein-protein interactions. Through the allosteric study of conformational changes in DNMT1 complexes, we have explored the characteristic indexes describing the allosteric process of mutation and PTM sites, so as to further explain the long-range allosteric pathways to its partner proteins. Underling the computational framework by integrating biophysical and bioinformatics methods, we describe the functional sites qualitatively and quantitatively, so as to apply the research of pocket pathway analysis to guide PPI-based allosteric drug design for DNA methylation. As a pure computational study, the predicted allosteric sites, signaling mutations, and PTMs present viable candidates for further experimental testing and validation. In the future, we hope that our computational results can be linked by experimental data such as from HDX-MS analysis or cross-linking mass spectrometry.

## Data Availability

Not applicable.

## Supplementary Materials

The following are available online, Figure S1: The motions of (A) the first and (B) the third ANM modes for Apo-DNMT1, Figure S2: Conformation dynamics of DNMT1 in three different states. The comparison of the (A) RMSDs and (B) RMSFs for Apo-DNMT1 (black lines), H3Ub-bound (green lines), and USP7-bound (red lines) states, Figure S3: PRS maps for (A) Apo-DNMT1, (B) DNMT1-H3Ub, and (C) DNMT1-USP7 complexes, Figure S4: (A) Pocket 1 and (B) pocket 5 for DNMT1-H3Ub, Figure S5: (A) Pocket 1 and (B) pocket 2 for DNMT1-USP7, Table S1: The studied disease mutations in DNMT1, Table S2: The studied PTM sites in DNMT1, Table S3: Allosteric pathways in DNMT1-H3Ub/USP7 complexes, Table S4: The allosteric binding properties, scoring functions, and constituting residues for detected allosteric sites in DNMT1-H3Ub, Table S5: The allosteric binding properties, scoring functions, and constituting residues for detected allosteric sites in DNMT1-USP7.

## Sample Availability

Not available.
