# Supplementary material for: Insights into Conformational Dynamics and Allostery in DNMT1-H3Ub/USP7 Interactions
Source: Molecules. 2021 Aug 25;26(17):5153. doi: 10.3390/molecules26175153 (PMC8434485; doi:10.3390/molecules26175153)
Supplement: Supplementary file 1 [file molecules-26-05153-s001.zip › Supplementary File/Supporting Informtaion.pdf]

Supporting Information

Insights into Conformational Dynamics and Allostery in DNMT1–H3Ub/USP7 Interactions

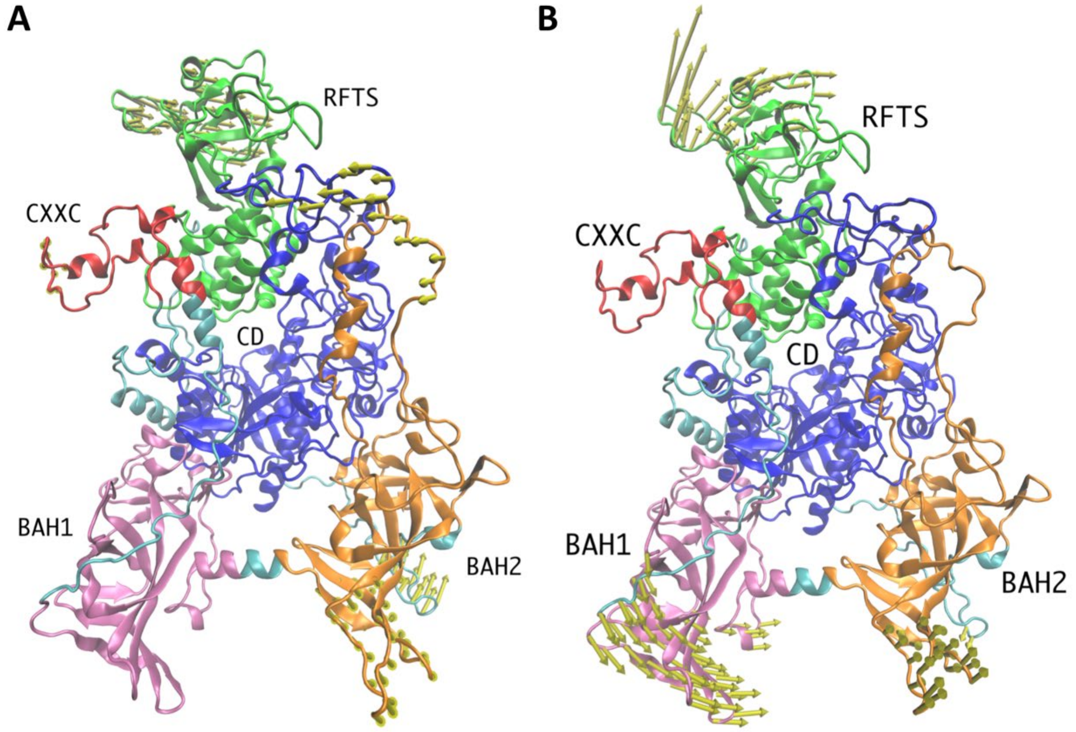

Figure S1. The motions of (A) the first and (B) the third ANM modes for the Apo-DNMT1.

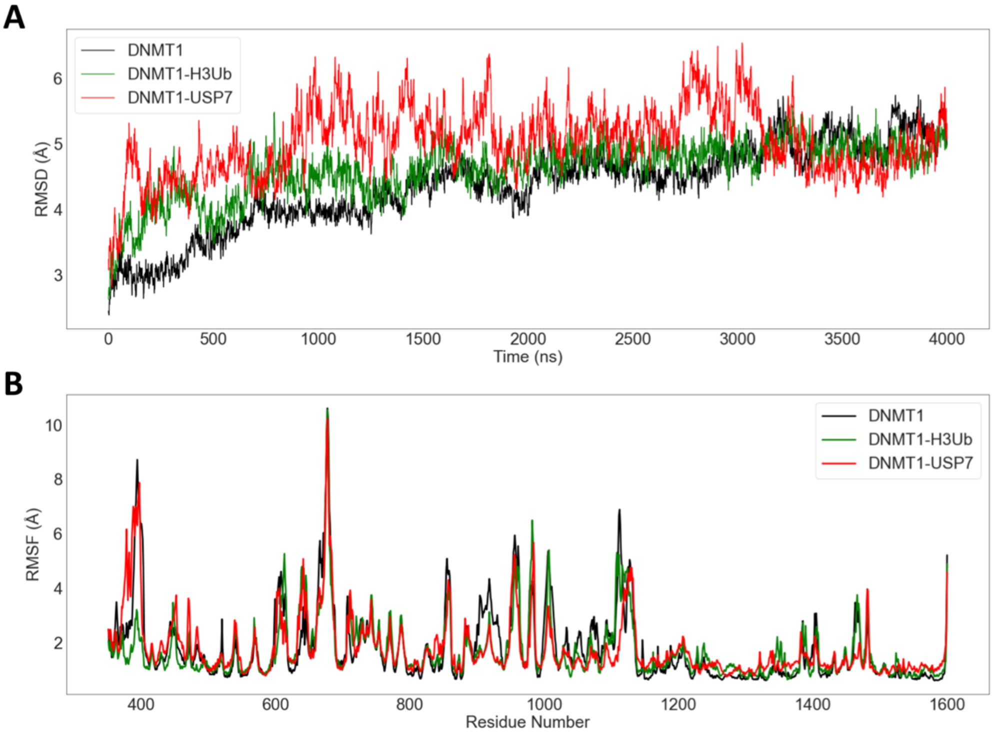

Figure S2. Conformation dynamics of DNMT1 in three different states. The comparison of the (A) RMSDs and (B) RMSFs for Apo-DNMT1 (black lines), H3UB-bound (green lines), and USP7-bound (red lines) states.

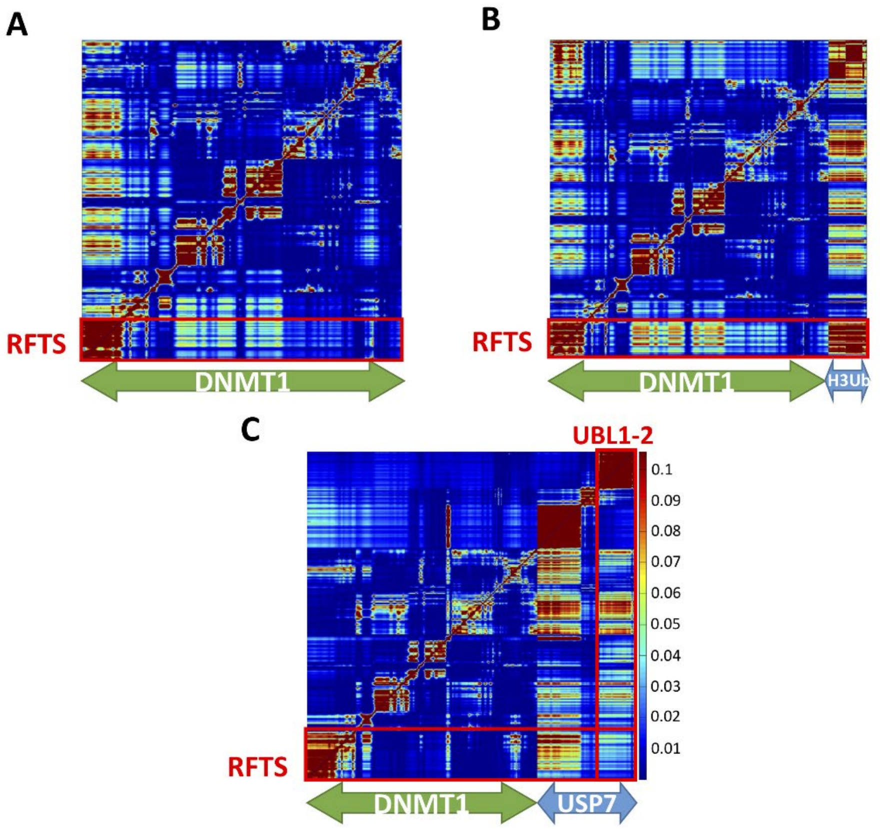

Figure S3. PRS maps for (A) Apo-DNMT1, (B) DNMT1-H3Ub, and (C) DNMT1-USP complexes. Regions marked by red rectangles highlight that RFTS domain in DNMT1 and UBL1-2 domain in USP7 has the most allosteric propensities.

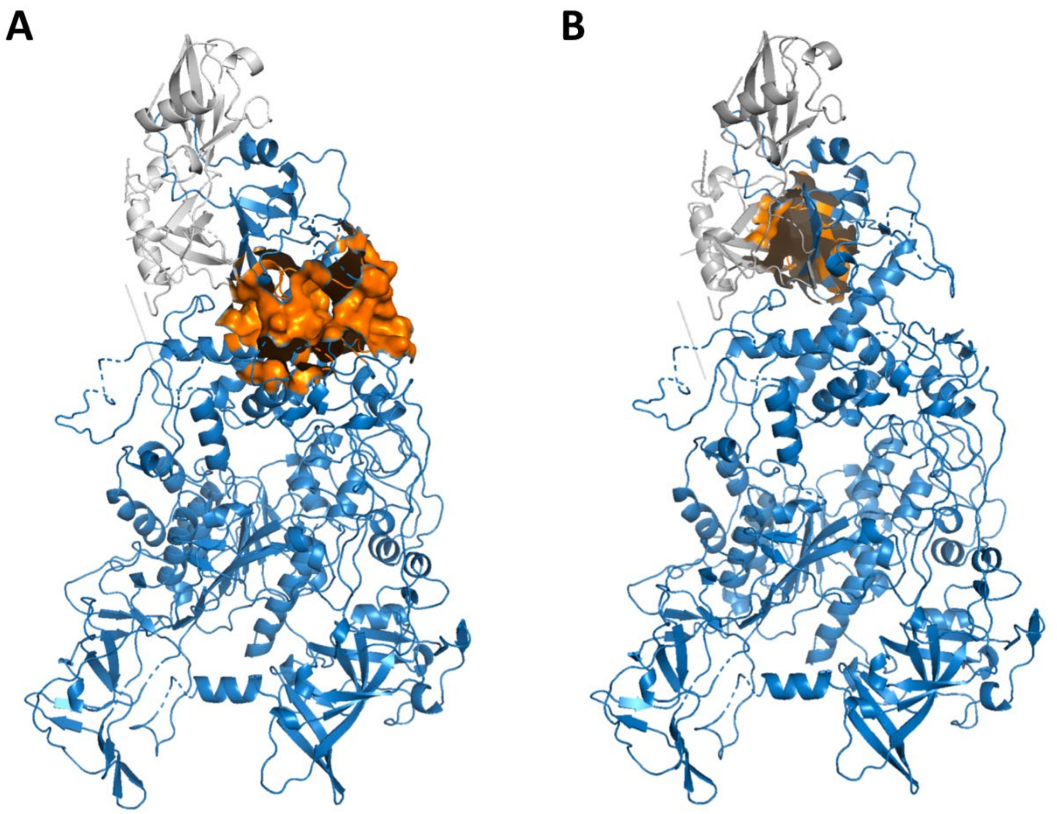

Figure S4. (A) The pocket 1 and (B) the pocket 5 for the DNMT1-H3Ub.

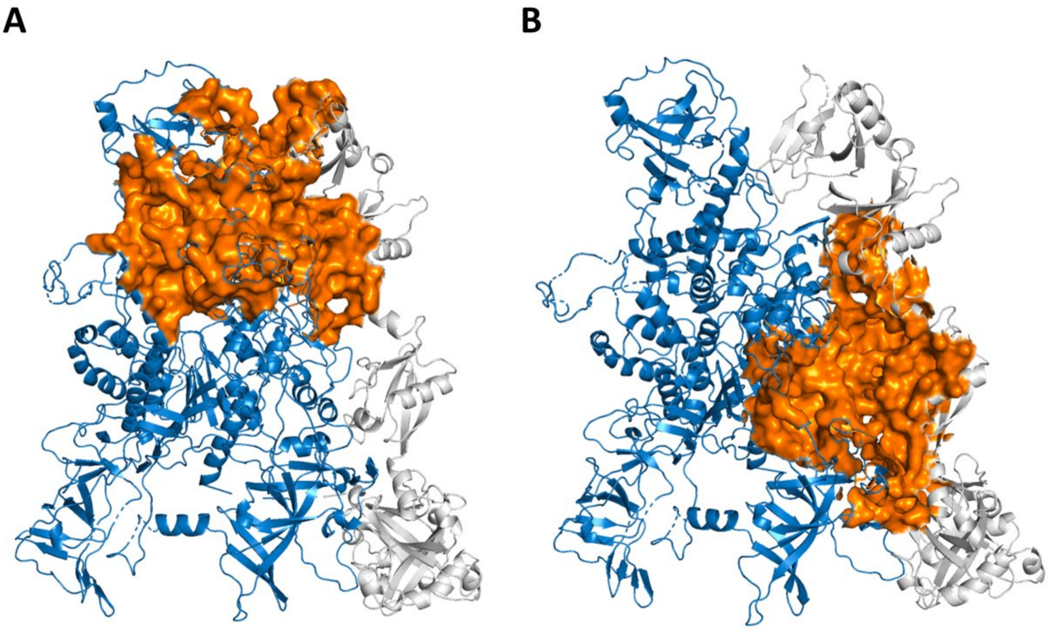

Figure S5. (A) The pocket 1 and (B) the pocket 2 for the DNMT1-USP7.

Table S1. The studied disease mutations in DNMT1.

Mutations in diseases	Diseases
Cys353Phe, Thr481Pro, Asp490Glu, Pro491Tyr/Leu/Arg, Tyr495Cys/His, Lys505del, Tyr524Asp, Ile531Asn, His553Arg	hereditary sensory and autonomic neuropathy with dementia and hearing loss type IE (HSAN IE)
Ala554Val, Cys580Arg, Gly589Ala, Val590Phe	autosomal dominant cerebellar ataxia, deafness and narcolepsy (ADCA-DN)

Table S2. The studied PTM sites in DNMT1.

Residue	Domain	PTM Type
Lys366	RFTS	Acetylation
Lys675	CXXC	Ubiquitylation
Ser714	linker	Phosphorylation
Lys739	BAH1	Sumoylation
Lys749	BAH1	Sumoylation
Lys891	BAH1	Acetylation
Lys957	BAH2	Acetylation/Sumoylation
Lys961	BAH2	Acetylation
Lys975	BAH2	Acetylation
Lys1054	BAH2	Acetylation
Lys1111	KG repeat	Acetylation
Lys1113	KG repeat	Acetylation
Lys1115	KG repeat	Acetylation
Lys1117	KG repeat	Acetylation
Lys1135	KG repeat	Sumoylation
Lys1349	CD	Acetylation
Lys1415	CD	Acetylation

Table S4. The allosteric binding properties, scoring functions and constituting residues for detected allosteric sites in DNMT1- H3Ub.

Allosteric Pockets	Pocket Volume (Å³)	Total SASA (Å²)	Druggability Score	Residues
**1**	3052.184	1483.075	0.189	A.351、A.356、A.357、A.358、A.359、A.361、A.362、A.363、A.364、A.365、A.366、A.418、A.419、A.421、A.422、A.423、A.424、A.425、A.426、A.427、A.428、A.464、A.494、A.497、A.498、A.501、A.505、A.551、A.552、A.553、A.554、A.555、A.556、A.559、A.588、A.589、A.590、A.1443、A.1451、A.1453、A.1454、A.1455、A.1456、A.1458、A.1475、A.1476、A.1485、A.1486、A.1487、A.1488、A.1489、A.1490、A.1491、A.1492、A.1493、A.1494、A.1498、A.1502<br><br>D.12、D.13
**2**	3232.796	1387.347	0.079	A.515、A.518、A.519、A.520、A.521、A.522、A.523、A.524、A.525、A.526、A.528、A.529、A.531、A.532、A.533、A.543、A.544、A.545、A.546、A.547、A.548、A.549、A.552、A.1227、A.1413、A.1414、A.1415、A.1416、A.1417、A.1418、A.1419、A.1420、A.1421、A.1446、A.1448、A.1450、A.1451、A.1452、A.1493、A.1514、A.1528、A.1529、A.1530、A.1531、A.1532、A.1533、A.1534、A.1547、A.1548、A.1550、A.1568、A.1569、A.1570、A.1571、A.1573、A.1574
**3**	771.148	356.515	0.286	A.466、A.467、A.468、A.469、A.506、A.509、A.510、A.513、A.556、A.560、A.564、A.599、A.601、A.602、A.603<br><br>D.10、D.11、D.12
**4**	3761.004	1485.373	0.465	A.378、A.380、A.381、A.382、A.383、A.384、A.386、A.387、A.388、A.390、A.391、A.392、A.393、A.394、A.395、A.396、A.397、A.400、A.401、A.402、A.403、A.404、A.435、A.439、A.442、A.457、A.459、A.481、A.483、A.484、A.485、A.486<br><br>B.42、B.44、B.70、B.71、B.72、B.73、B.74

				C.6、C.7、C.8、C.10、C.12、C.13、C.30、C.35、C.36、C.37、C.40、C.44、C.49、C.68、C.69、C.70、C.71、C.73
**5**	846.021	399.205	0.375	A.404、A.406、A.442、A.443、A.444、A.445、A.467、A.470、A.474、A.476、A.477、A.487、A.488、A.489<br><br>B.6、B.7、B.8、B.9、B.10、B.44、B.68、B.69、B.70、B.71、B.73

Table S5. The allosteric binding properties, scoring functions and constituting residues for detected allosteric sites in DNMT1- USP7.

Allosteric Pockets	Pocket Volume (Å^3)	Total SASA (Å^2)	Druggability Score	Residues
**1**	13279.57	7053.9	0.103	A.352、A.356、A.357、A.358、A.359、A.361、A.362、A.363、A.364、A.365、A.366、A.367、A.368、A.369、A.370、A.371、A.409、A.410、A.411、A.412、A.413、A.417、A.419、A.420、A.421、A.422、A.423、A.424、A.425、A.426、A.427、A.428、A.429、A.430、A.436、A.456、A.457、A.462、A.463、A.464、A.465、A.477、A.488、A.492、A.493、A.494、A.495、A.496、A.497、A.498、A.499、A.500、A.501、A.502、A.504、A.505、A.528、A.531、A.532、A.536、A.539、A.540、A.541、A.542、A.543、A.544、A.545、A.546、A.547、A.548、A.549、A.550、A.552、A.553、A.555、A.556、A.558、A.559、A.562、A.585、A.586、A.589、A.590、A.591、A.592、A.593、A.594、A.595、A.596、A.597、A.598、A.599、A.600、A.653、A.655、A.656、A.657、A.660、A.693、A.696、A.697、A.700、A.966、A.967、A.970、A.971、A.973、A.974、A.1376、A.1377、A.1378、A.1379、A.1415、A.1416、A.1417、A.1418、A.1419、A.1420、A.1421、A.1422、A.1423、A.1424、A.1425、A.1426、A.1427、A.1430、A.1441、A.1443、A.1444、A.1445、A.1446、A.1447、A.1448、A.1449、A.1450、A.1451、A.1453、A.1454、A.1455、A.1456、A.1457、A.1459、A.1460、A.1461、A.1462、A.1463、A.1464、A.1467、A.1469、A.1471、A.1472、A.1473、A.1474、A.1475、A.1476、A.1477、A.1478、A.1479、A.1481、A.1482、A.1483、A.1484、A.1485、A.1486、A.1487、A.1488、A.1489、A.1490、A.1491、A.1492、

				A.1493、A.1494、A.1501、A.1502、A.1503、A.1505、A.1506、A.1507、A.1514、A.1531、A.1532、A.1533、A.1543、A.1544、A.1545、A.1546<br><br>B.892、B.914、B.915、B.916、B.943、B.945、B.946、B.948、B.950、B.951、B.952、B.953、B.954、B.956、B.957、B.958、B.959、B.960、B.961、B.962、B.963、B.967、B.968、B.999、B.1000、B.1002、B.1016、B.1017、B.1018、B.1021、B.1038、B.1039、B.1040、B.1042、B.1043、B.1044、B.1045、B.1046、B.1047、B.1048、B.1049、B.1050、B.1051、B.1052、B.1053、B.1055、B.1056、B.1057、B.1058、B.1059、B.1061、B.1062、B.1063、B.1065、B.1066、B.1067、B.1068、B.1069、B.1070、B.1071、B.1072、B.1073、B.1074、B.1075、B.1076
**2**	13955.021	6901.137	0.137	A.954、A.955、A.956、A.957、A.980、A.981、A.982、A.983、A.984、A.985、A.986、A.987、A.988、A.989、A.990、A.1023、A.1024、A.1025、A.1026、A.1027、A.1029、A.1031、A.1032、A.1033、A.1034、A.1035、A.1036、A.1037、A.1038、A.1039、A.1040、A.1041、A.1043、A.1044、A.1045、A.1046、A.1064、A.1065、A.1066、A.1071、A.1072、A.1073、A.1074、A.1076、A.1077、A.1078、A.1079、A.1080、A.1082、A.1084、A.1086、A.1087、A.1088、A.1089、A.1090、A.1096、A.1097、A.1098、A.1099、A.1100、A.1101、A.1104、A.1131、A.1132、A.1133、A.1156、A.1157、A.1158、A.1159、A.1304、A.1305、A.1309、A.1331、A.1332、A.1333、A.1335、A.1362、A.1363、A.1364、A.1365、A.1366、A.1368、A.1369、A.1372、A.1373、A.1374、A.1375、A.1376、A.1377、A.1378、A.1381、A.1382、A.1383、A.1386、A.1387、A.1388、A.1389、A.1390、A.1391、A.1392、A.1393、A.1394、A.1395、A.1396、A.1398、A.1399、A.1400、A.1401、A.1402、A.1403、A.1404、A.1405、A.1406、A.1408、A.1409、A.1410、A.1432、A.1517、A.1518、A.1519、A.1520、A.1521、A.1522、A.1523、A.1524、A.1525、A.1538、A.1543、A.1544、A.1551、A.1558、A.1560、A.1561、A.1562、A.1564、A.1582、A.1583、A.1586、A.1587、A.1590、A.1593<br><br>B.581、B.737、B.739、B.741、B.742、B.743、B.784、B.787、B.788、B.791、B.792、B.794、B.796、B.815、B.816、B.818、B.819、B.820、B.823、B.824、B.833、B.834、B.835、B.837、B.838、B.839、B.841、B.842、B.843、B.844、B.845、B.846、B.847、B.848、B.851、B.852、B.853、B.854、B.855、B.856、B.857、B.858、B.859、B.860、B.862、B.863、B.864、B.865、

				B.866、B.867、B.868、B.869、B.870、B.872、B.873、B.874、B.875、B.876、B.877、B.885、B.886、B.888、B.889、B.891、B.892、B.893、B.894、B.895、B.896、B.897、B.907、B.911、B.912、B.913、B.914、B.959、B.964、B.965、B.966、B.968、B.969、B.970
**3**	3064.893	1318.425	0.017	A.525、A.526、A.529、A.532、A.533、A.544、A.1145、A.1147、A.1167、A.1168、A.1169、A.1170、A.1172、A.1173、A.1188、A.1189、A.1190、A.1191、A.1223、A.1224、A.1225、A.1247、A.1266、A.1267、A.1268、A.1269、A.1310、A.1311、A.1312、A.1380、A.1381、A.1382、A.1384、A.1413、A.1414、A.1415、A.1416、A.1525、A.1528、A.1529、A.1568、A.1569、A.1570、A.1571、A.1574、A.1575、A.1577、A.1578<br><br>B.965、B.966、B.967
